# A research on urban disaster resilience assessment system for rainstorm and flood disasters: A case study of Beijing

**DOI:** 10.1371/journal.pone.0291674

**Published:** 2023-10-26

**Authors:** Shuangrui Yu, Ruiqi Li, Yuxi Zhang, Mingfei Wang, Peng Zhang, Aizhi Wu, Fucai Yu, Xiaofeng Zhang, Lin Yang, Yong’an Cui

**Affiliations:** 1 Beijing Academy of Emergency Management Science and Technology, Beijing, 101117, China; 2 China Academy of Urban Planning and Design, Beijing, 100044, China; 3 Beijing Institute of Astronautical System Engineering, Beijing, 100076, China; 4 Beijing Xiaoban Technology Co., Beijing, 100020, China; 5 Institute of Urban Safety and Environmental Science, Beijing Academy of Science and Technology, Beijing, 100054, China; 6 Emergency Rescue Center (Fire Brigade) of Dongying City, Shandong Province, Dongying, 257000, China; University of Strathclyde, UNITED KINGDOM

## Abstract

Under the background of global climate change, rainstorm and flood disasters have become the most serious cataclysm. Under the circumstances of an increasingly severe risk situation, it is necessary to enhance urban disaster resilience. Based on the disaster resilience process of prevention, absorption, and enhancement, and considering the safety factors such as personnel, facility, environment and management, this paper forms a dual dimension of the urban disaster resilience assessment model covering the key elements of urban disaster response and the core capacity of urban disaster recovery. Furthermore, if taking into account the characteristics of rainstorm and flood disasters, the paper screens the key indicators to build up an assessment index system of an urban rainstorm and flood disaster. The practical application was implemented in Beijing to have an assessment of the ability to recover from rainstorm and flood disasters in all districts of Beijing. And then, some pertinent suggestions for enhancing the resilience of Beijing to rainstorm and flood disasters were proposed.

## 1 Introduction

With the ceaseless proceeding of global warming, the accelerating regional water circulation leads to a high risk of extreme rainfall. Hence, flood disaster caused by rainstorm has been one of the dominant threats faced by modern cities. According to relevant research, there are currently 1.81 billion people in the world directly exposed to the threat of once-in-a-century flood disasters, including 1.24 billion people in East and South Asia, and China (395 million) accounts for the highest risk share [1]. On July 20, 2021, a flood disaster caused by extreme rainfall made major deaths and property losses in Zhengzhou City, Henan Province, China, which raised a great repercussions in the local society.

In an increasingly complex and severe disaster risk environment, improving urban disaster resilience has become a public topic of concern. The United Nations defines resilience as the ability of a system, community or society exposed to disasters to resist, absorb, accommodate and recover from the effects of a hazard in a timely and efficient manner, including through the preservation and restoration of its essential basic structures and functions [2]. The ISO/TC 292 Technical Committee on Safety and Resilience defines urban resilience to be the ability of any urban system, with its inhabitants, in a changing environment, to anticipate, prepare, respond to and absorb shocks, positively adapt and transform in the face of stresses and challenges, while facilitating inclusive and sustainable development [3]. It could be seen that disaster resilience emphasizes the city’s ability to resist and respond to disasters in the whole process of pre-disaster prevention, in-disaster absorption and post-disaster enhancement. Cities such as New York [4], London [5], and Rotterdam [6], have also carried out actions to improve urban disaster resilience against climate change.

Scientists and researchers have widely focused on the way to enhance urban resilience for rainstorm and flood disasters. Aerts et al. set up a management strategy assessment method for the coastal big cities’ resilience to flood disasters from the aspect of cost-benefit [7]. Bertilsson et al. proposed the multiple standard indexes plotting method for urban resistance for flood disasters [8]. One of the priorities to formulate the public policy to enhance metropolitan strength for rainstorm and flood disasters is to have a quantitative assessment of them. One of the main methods to assess this is setting up a scenario of rainstorm and flood disasters through the meteorological and hydrological models to analyze the exposure and vulnerability of disaster-bearing bodies. For example, Tayyab et al. set up an assessment model of municipalities’ recovery after downpour catastrophes based on the geographic information system and remote sensing data, which has a comprehensive thought of such influencing factors as the flood disaster risk, coping ability, and conduct the application in the city Bavasha, Pakistan [9]. Bisht et al. have studied the flooding process under extreme precipitation times in a region of West Bengal, India with the models of SWMM and MIKE [10]. However, the method of simulation assessment based on the meteorological and hydrological measures would need a large amount of data on the precipitation model, ground elevation, pipe network distribution and so on, and the analysis would need to be organized with drainage zoning of small areas as the basic unit. The large-scale application of the urban regional hierarchy would be restricted by insufficient basic data. The other method for the disaster risk and resilience assessment is to estimate based on the index system, whose advantage is to ponder on multiple factors and to have a flexible operation. This method has been applied in many resilience assessments on disasters related to climate change, such as drought disasters, sea ice disasters, and hurricane disasters [10–13]. Some researchers also carry out studies on the assessment system of resilience for rainstorm and flood disasters. For example, Ali et al. built up assessment principles from society, economy, politics, health, communication, education, infrastructures, and other dimensions to propose a community resilience index system in flood-prone areas of the city [14]. Balica et al. developed the flood vulnerability index system of coastal cities from the perspectives of exposure, vulnerability, and resistance, then selected 9 cities worldwide for evaluation and application [15]. It should be noted that researchers mainly proceed through two ways when constructing evaluation systems and presenting results in existing studies related to system resilience assessment. One focuses more on resilience, portraying the similarities and gaps between the system and an ideal resilient system through the description of the system’s resilience capabilities or characteristics [16–18]. The other focuses more on the system, sorting out the potential specific influences through the system’s component dimensions [19–21]. These two ways can be considered as two perspectives of considering system resilience and have been mentioned in the early literature about resilience studies [22]. However, considering the system resilience from only one way of the two above may have some impact on the study. Studying only from the perspective of system’s resilience capabilities or characteristics, the indicators are limited by and the comprehensiveness and precision of the capabilities or characteristics. And the descriptions of capabilities or characteristics are generally abstract, so it is not intuitive enough to assess how to enhance system resilience through practical work based on the assessment results. Studying only from the perspective of system’s components, it is easily lead to conceptual confusion between resilience research and traditional risk research for the lack of interpretation of what resilience is. Therefore, this paper proposes the assessment model with the dual dimensions of urban disaster resilience which integrates the two perspectives. In the empirical evaluation of disaster resilience in Beijing, in addition to the overall disaster resilience assessment, this paper further carries out separate assessments from the two perspectives in order to interpret system resilience more comprehensively.

Section 1 of this paper introduces the research background and analyzes the research status of urban resilience assessment for a rainstorm and flood disasters. Section 2 proposes an assessment model with the dual dimensions of urban disaster resilience with a comprehensive consideration of the MMEM (Man-Machine-Environment-Management) theory model and resilience process. Section 3 sets up an assessment index system of urban rainstorm and flood disasters with 3 level-2 indicators, 11 level-3 indicators and 31 level-4 indicators, to provide a tool for the examination of the urban resilience of rainstorm and flood disasters. Section 4 takes Beijing as the research object, determines the weights of indicators through the analytic hierarchy process, and carries out the application of rainstorms and the ability to estimate recovery from floods for each district of Beijing based on the government department data. Section 5 analyzes the general situation of the resilience of rainstorm and flood disasters in all districts of Beijing, as well as the various strength capabilities and safety factors, analyze the existing problems and proposes the improvement direction to have a comparative analysis of the resilience results in the central areas and the non-central areas. Section 6 is to make a summary of the study contents and the conclusion of this paper.

## 2 Theoretical model for urban disaster resilience assessment

For carrying out the urban disaster resilience assessment, it is necessary to establish the assessment dimensions and framework. Researchers mainly have two ideas when setting up the resilience assessment dimensions currently. The first idea is to begin with the principle to evaluate the several core abilities embodied in the cities during the disaster response process. The conduction of the assessment of urban disaster resilience based on this idea would be to understand the main tasks faced by cities in different disaster response stages, but there would be difficulty in the explanation of the internal logic between the sorted indicators. If it will fail to correspond to the specific responsibilities of the urban management departments, it would be harmful to the city to generate the actual strategy to enhance disaster resilience. The second idea is to conduct based on several key factors of the urban disaster response to have an assessment from the perspective of the system composition. Based on this idea, urban disaster resilience assessment is apt to integrate abundant kinds of factors involved and sort out the assessment indicators, but it is hard to explain the role of various elements in different stages of urban disaster response. Also, it would be difficult to highlight the resilience concept and the essence reflected in the traditional disaster vulnerability assessment.

**Fig 1:**
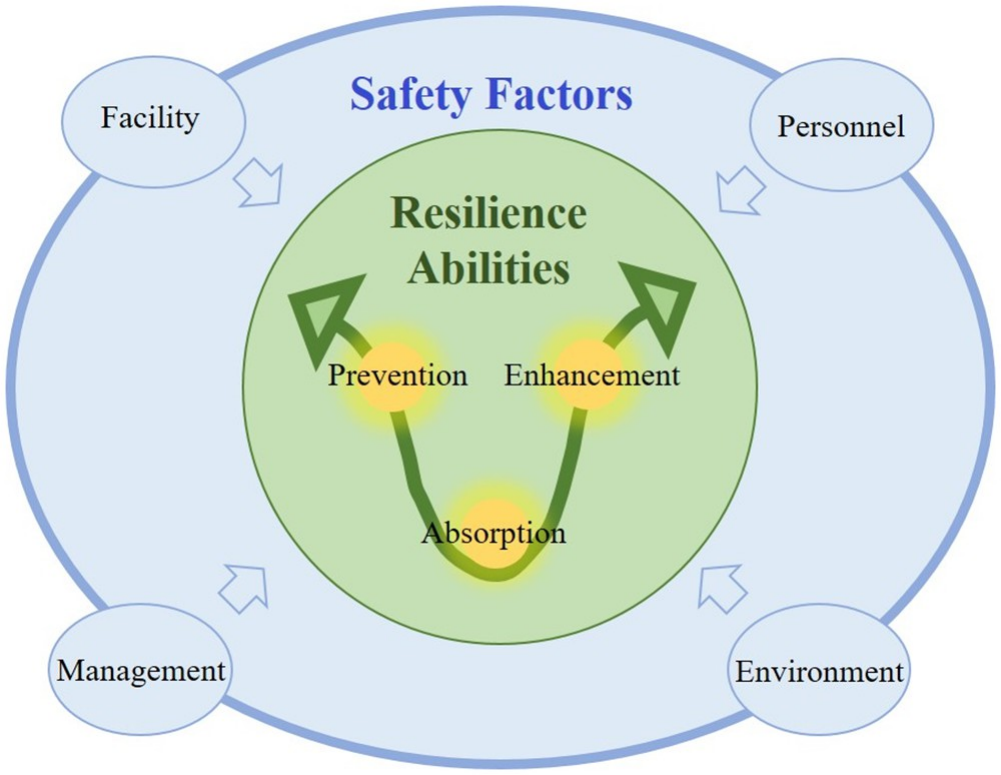
Assessment model with the dual dimensions of urban disaster resilience.

To solve the problems mentioned above, better to build an assessment of the index system of urban disaster resilience with two dimensions, the core abilities of resilience and the key factors of urban disaster response. In addition, to provide support for the comprehensive and systemic assessment of metropolitan disaster resilience, there is a decoupling of the connotative relation on all indicators and the two assessment dimensions. Consequently, the paper proposes the following examination model with dual dimensions (as shown in Fig 1). In this model, there are two assessment vectors, including resilience abilities and safety factors.

There are different summarizations for the dimension of resilience abilities. It is generally believed that three main theories exist for the perception of the concept of resilience, namely, engineering resilience, ecological resilience, and socio-ecological resilience [23]. These three theories emphasize the characteristics that a resilient system should have. Engineering resilience is the earliest of the three perspectives. It refers to the ability of the system to minimize losses in order to maintain and recover as soon as possible the original [24]. It could be found that the theory of engineering resilience emphasizes the system should be as stable as possible. Thus prevention ability should be taken as a core ability of resilience. Ecological resilience emphasizes more on the magnitude of disturbance that a system can absorb before structural change, and emphasized the existence of multi-stability of the system [25]. From this theory, absorption ability should also be taken as a core ability of resilience. Then Walker et al. proposed the concept of socio-ecological resilience, which emphasis on the system’s ability to adapt and enhance in a constantly changing environment [26]. It could be found that enhancement ability is a core ability of resilience as well. Therefore, the paper concludes the resilience abilities into prevention, absorption and enhancement of these three aspects from a whole process of urban disaster response, including pre-disaster, in-disaster and post-disaster. The definitions are given with reference to other literature [18]. Prevention indicates the ability of a city to reduce the possibility of adverse consequences of disasters [27]. It takes place through various measures before the arrival of disaster events. Absorption means the ability of a city to minimize the damage to itself caused by a disaster event when it occurs [28]. Enhancement refers to the ability of the city to recover from the impact of disaster events as soon as possible and learn from them to reduce future disaster risks [29]. These three kinds of resilience abilities reflect various concerns and the requirements for enhancing urban cataclysm resilience.

Different researchers also proposed different perspectives on the analytical dimensions of safety factors. For example, Bruneau et al. conceptualized the factors as four interrelated dimensions: technical, organizational, social, and economic [22]. However, in order to present separate resilience evaluation results from each safety factor dimension, the division among factors needs clearer boundaries. The factors need to be less interrelated to ensure that the evaluation results could reflect the resilience characteristics of a certain aspect of the system elements independently. It will be more helpful for clarifying the direction of resilience enhancement. The MMEM is a theoretical model of risk management with guiding significance, which is widely applied to various kinds of risk management and safety accidents [30]. This model defines safety factors into four relatively independent fields, including man, machine, environment and management. The mutual impact and joint working of the safety factors are reflected by the chain angle of safety risk breedings, controls and disaster generating. The study object of urban disaster resilience assessment is the urban system. Compared with the specific safety incident risk management, the study object would be wider in the space scale, and the causes of risk are more complicated, so there should be much more consideration of the roles of various kinds of safety factors in urban disaster resilience. The paper applies the MMEM theory model to the study of urban disaster resilience and concludes the four influencing safety factors as personnel, facility, environment and management.

## 3 The assessment index system of urban resilience for rainstorm and flood disasters

According to the assessment model with the dual dimensions of urban disaster resilience mentioned above, the paper first analyzes the three aspects of the dimension of resilience abilities, prevention resilience, absorption resilience and enhancement resilience, and analyzes the leading factors in the process of urban rainstorms and flood disaster response.

In the aspect of prevention resilience, factors including land layout, monitoring and early warning, and information release are mainly considered [31–33]. The land layout affects the exposure and vulnerability of urban disaster-bearing bodies, which is the premise to reduce the physical loss caused by rainstorm and flood disasters. The enhancement of monitoring and early warning capability could precisely control the process of disaster breeding, development and extinction to achieve more effective disaster prevention and mitigation. The information release is a crucial link to guide the social mass to have disaster prevention and mitigation, which plays a key role in reducing the casualties due to disasters.

In terms of absorption resilience, factors including natural environment, engineering resistance, evacuation and risk avoidance, and social response are mainly considered [34,35]. The natural environment means the background of urban blue and green space [36]. Rich blue and green space could effectively absorb and weaken the strength of rainstorm and flood disasters. The engineering resistance is to protect the important disaster-bearing bodies through the way of engineering to reduce the vulnerability of disaster-bearing bodies [37]. Evacuation and risk avoidance refer to protecting the people threatened by disasters through transfer and temporary refuge in the case of disasters exceeding the fortification intensity [38]. Social response mainly shows the vulnerability of the urban population and the ability in medical aid, emergency rescue and other aspects [39,40].

In terms of enhancement resilience, factors including risk management, financial input, community preparation and social recovery are mainly considered [41–43]. Risk management primarily refers to the ability of cities to reduce casualties and economic losses caused by disasters, which is the embodiment of the comprehensive level of urban risk management [44]. The financial input is the capital invested by cities in risk prevention and post-disaster recovery, which is an essential guarantee to enhance urban healing from the cataclysm [45]. Community preparation emphasizes the disaster response ability of the grassroots community, which is the necessary fundamental job implemented to enhance urban disaster resilience [46]. Social recovery mainly indicates the ability of cities to recover quickly from the impact of disasters, which is the core requirement of metropolitan ability to resist and recover from cataclysm [47].

Furthermore, this paper selects 31 specific quantitative evaluation indicators based on the above factors, considering the availability and comparability of data and fully combining them with the government’s disaster prevention and emergency functions. These indicators are available in the public data statistics of government sectors. Ulteriorly, the paper has a classification of factors for the assessment dimension of safety factors to clarify the correspondent relation of all indicators with the four safety factors, personnel, facility, environment and management, as shown in Table 1. And the the data source sectors are also presented.

**Table 1:** Assessment index system of urban resilience for rainstorm and flood disasters.

Level-1 Indicator	Level-2 Indicator	Level-3 Indicator	Level-4 Indicator	Factor Classification	Indicator Direction	Data Source Sector
Urban resilience for rainstorm and flood disasters	C_1_ Prevention resilience	T_1_ Land Layout	I_1_ Land Development Intensity	Environment	Negative Direction	Housing and Urban-Rural Development Sector
			I_2_ Proportion of Water and Soil Loss Control Area	Environment	Positive Direction	Water Resources Sector
			I_3_ Proportion of Water Area and Water Conservancy Facilities	Facility	Positive Direction	Natural Resources Sector
			I_4_ Proportion of land for transportation facilities	Facility	Positive Direction	Natural Resources Sector
		T_2_ Monitoring and early warning	I_5_ Early warning amount of sudden disastrous weather	Facility	Positive Direction	Meteorological Sector
			I_6_ Number of Disaster Information Officers per 10,000 People	Personnel	Positive Direction	Emergency Management Sector
		T_3_ Information Release	I_7_ Mobile phone penetration	Facility	Positive Direction	Industry and Information Technology Sector
	C_2_ Absorption resilience	T_4_ Natural environment	I_8_ Annual rainfall	Environment	Negative Direction	Meteorological Sector
			I_9_ Reservoir density	Environment	Positive Direction	Water Resources Sector
			I_10_ Greening coverage	Environment	Positive Direction	Housing and Urban-Rural Development Sector
		T_5_ Engineering resistance	I_11_ Pump station density	Facility	Positive Direction	Water Resources Sector
			I_12_ Comprehensive rainwater utilization capacity	Facility	Positive Direction	Water Resources Sector
			I_13_ Proportion of urban built-up areas that can absorb and utilize 70% of rainfall locally	Facility	Positive Direction	Housing and Urban-Rural Development Sector
			I_14_ Dike density	Facility	Positive Direction	Water Resources Sector
		T_6_ Evacuation and Risk Avoidance	I_15_ Road area per capita	Facility	Positive Direction	Housing and Urban-Rural Development Sector
			I_16_ Road traffic index	Facility	Negative Direction	Transport Sector
			I_17_ Number of private cars per 100 households	Facility	Positive Direction	Public Security Sector
			I_18_ Area of refuge per capita	Facility	Positive Direction	Emergency Management Sector
		T_7_ Social response	I_19_ Permanent population density	Personnel	Negative Direction	Statistics Sector
			I_20_ Proportion of young and old people	Personnel	Negative Direction	Statistics Sector
			I_21_ Number of health technicians per 10,000 people	Personnel	Positive Direction	Health Sector
			I_22_ Number of emergency rescue volunteer registered groups per 10,000 people	Personnel	Positive Direction	Civil Affairs Sector
			I_23_ Proportion of water conservancy, environment and public facilities management in employment	Personnel	Positive Direction	Human Resources and Social Security Sector
	C_3_ Enhancement resilience	T_8_ Risk management	I_24_ Proportion of the affected population	Personnel	Negative Direction	Emergency Management Sector
			I_25_ Proportion of direct economic losses caused by disasters to GDP	Management	Negative Direction	Emergency Management Sector
		T_9_ Financial input	I_26_ Proportion of public security expenditure	Management	Positive Direction	Finance Sector
			I_27_ Proportion of medical and health expenditure	Management	Positive Direction	Finance Sector
		T_10_ Community preparation	I_28_ Proportion of comprehensive disaster reduction demonstration communities	Management	Positive Direction	Emergency Management Sector
			I_29_ Number of community service agencies per 10,000 population	Management	Positive Direction	Civil Affairs Sector
		T_11_ Social recovery	I_30_ Proportion of the population with basic medical insurance	Personnel	Positive Direction	Human Resources and Social Security Sector
			I_31_ Disposable income per capita	Personnel	Positive Direction	Statistics Sector

Table 1 also shows the vector of each specific indicator, which is divided into positive indicators and negative indicators. The direction of the indicator is the directional correlation between data value and urban resilience for rainstorm and flood disasters. The larger the data value of the positive indicator, the stronger the urban resilience for rainstorm and flood disasters; while the smaller the data value of the negative indicator, the stronger the urban resilience for rainstorm and flood disasters.

## 4 Data processing and quantitative evaluation methods

### 4.1 Research object and data sources

Beijing is the capital of the People’s Republic of China with a division into 16 districts, which is a pioneer in the enhancement of disaster resilience among China’s cities. The paper made Beijing a study object to conduct an assessment of the resilience of rainstorm and flood disasters at the level of the district. With a comparison of the assessment index system of urban resilience for rainstorm and flood disasters, there was data collected from 16 districts. The data sources were the official statistics of relevant government departments in Beijing, and the year of data was 2020.

### 4.2 Data standardization

The data of all indicators were processed with deviation standardization [48].

For the positive indicators,

$$X_{i}=\frac{x_{i}-\text{min}\;x_{i}}{\text{max}\;x_{i}-\text{min}\;x_{i}}$$

For the negative indicators,

$$X_{i}=\frac{\text{max}\;x_{i}-x_{i}}{\text{max}\;x_{i}-\text{min}\;x_{i}}$$

In this formula, the *x*_*i*_ was the original data of the indicator and the *X*_*i*_ was the data after the standardization process.

### 4.3 Weight determination

Since the hierarchical relationships among indicators are clear, and the indicators in the same hierarchical model have strong comparability, so this study adopts with analytic hierarchy process (AHP) as the method of determining indicator weights. The AHP approach is considered to be an efficient and flexible framework based on psychology and mathematics and is thus an ideal subjective weighting method [48].

The steps of AHP approach are as follows.

**Fig 2:**
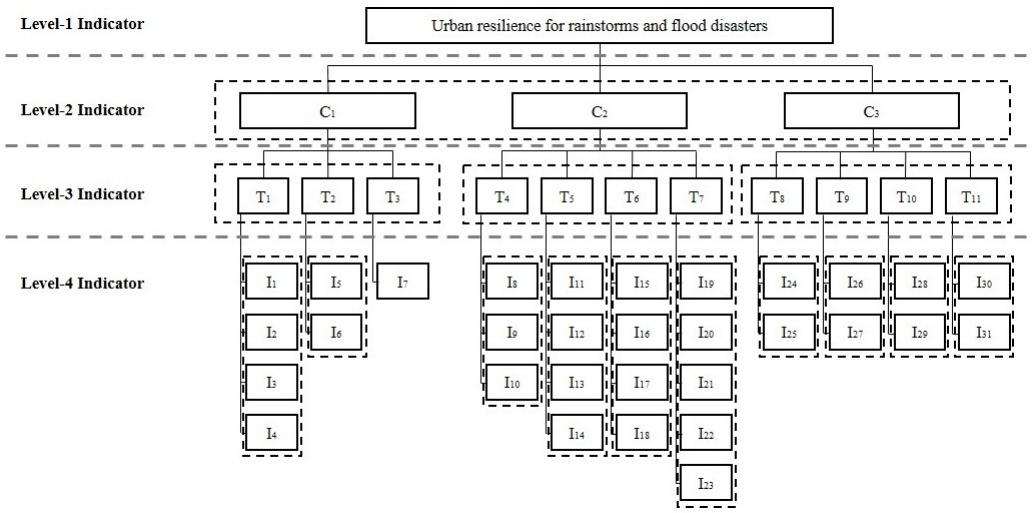
Hierarchical structure of assessment index system of urban resilience for rainstorm and flood disasters.

First, a hierarchical analogy based on the structure of the assessment index system needs to be established, as shown in Fig 2. The indicators represented by serial numbers in each dashed box in the Fig 2 are analogous objects of the same hierarchy.

Second, the importance of indicators in the same hierarchy are compared pair by pair and a judgment matrix is constructed. The relative importance scales are shown in Table 2.

**Table 2:** Correspondence between relative importance and scale quantification values.

Relative Importance of Indicator 1 to Indicator 2	Scale Quantification Value of Indicator 1 to indicator 2
Equally important	1
Slightly more important	3
Obviously more important	5
Strongly more important	7
Extremely more important	9
Between two adjacent descriptions above	2,4,6,8

The judgment matrix is a *n* × *n* matrix, in which *n* is the number of indicators in the same hierarchy. The element *a*_*ij*_ in the judgment matrix is the scale quantification value of indicator *i* to indicator *j*, and the following conditions need to be met.


$$a_{ij}=1\;(i=j)$$

$$a_{ij}=\frac{1}{a_{ji}}\;(i\neq j)$$

Third, the eigenvectors corresponding to the maximum eigenvalues of the judgment matrix are calculated as the relative weights of each indicator.

Fourth, a consistency test is needed to explore the discordances between the pairwise comparisons and the reliability of the obtained weights. The consistency test is conducted by calculating consistency ratio (*CR*), which is compared the consistency index (*CI*) with an given average random consistency index (*RI*). The calculation formulas are as follows.


$$CI=\frac{\lambda_{max}-n}{n-1}$$

$$CR=\frac{CI}{RI}$$

*λ*_max_ is the maximum eigenvalues of the judgment matrix, and *n* is the number of indicators in the hierarchy. *RI* is given by randomly generated matrices, which is determined by *n*.

Generally, 0.1 is adopted as the judgment value of *CR*, and a smaller result means better consistency among the subjective judgments.

Finally, the comprehensive weight of each indicator is calculated after the end of all the separate hierarchical analysis process.

Ten experts with different disciplinary backgrounds, such as emergency management, water conservancy, urban planning, remote sensing, and risk assessment were invited. And then it was adopted with an analytic hierarchy process to confirm the relative weight of all indicators at all levels, to gain the comprehensive weight of all specific assessment indicators. The weighted results of the indicators are shown in Table 3. The relative weights should be the judge of the relative importance of the indicators at the same level, which were rated by the experts. Only the results that pass the consistency test were retained. The results of average consistency ratio (*CR*) are shown in Table 4. The comprehensive weight was the judgement of the comprehensive importance of all specific assessment indicators, which was gained by the relative weight of the corresponding indicator and its superior indicator.

**Table 3:** Indicator weight results.

Level-1 Indicators	Relative Weight	Level-2 Indicators	Relative Weight	Level-3 Indicators	Relative Weight	Level-4 Indicators	Relative Weight	Comprehensive Weight
Urban resilience for rainstorm and flood disasters	1.00	C_1_ Prevention resilience	0.445	T_1_ Land layout	0.559	I_1_ Land development intensity	0.186	0.046
						I_2_ Proportion of water and soil loss control area	0.404	0.100
						I_3_ Proportion of water area and water conservancy facilities	0.257	0.064
						I_4_ Proportion of land for transportation facilities	0.153	0.038
				T_2_ Monitoring and early warning	0.232	I_5_ Early warning amount of sudden disastrous weather	0.747	0.077
						I_6_ Number of Disaster Information Officers per 10,000 People	0.253	0.026
				T_3_ Information Release	0.210	I_7_ Mobile phone penetration	1.000	0.093
		C_2_ Absorption resilience	0.333	T_4_ Natural environment	0.357	I_8_ Annual rainfall	0.389	0.046
						I_9_ Reservoir density	0.265	0.031
						I_10_ Greening coverage	0.346	0.041
				T_5_ Engineering resistance	0.283	I_11_ Pump station density	0.257	0.024
						I_12_ Comprehensive rainwater utilization capacity	0.153	0.014
						I_13_ Proportion of urban built-up areas that can absorb and utilize 70% of rainfall locally	0.404	0.038
						I_14_ Dike density	0.186	0.018
				T_6_ Evacuation and Risk Avoidance	0.188	I_15_ Road area per capita	0.245	0.015
						I_16_ Road traffic index	0.267	0.017
						I_17_ Number of private cars per 100 households	0.153	0.010
						I_18_ Area of refuge per capita	0.335	0.021
				T_7_ Social response	0.172	I_19_ Permanent population density	0.064	0.004
						I_20_ Proportion of young and old people	0.254	0.015
						I_21_ Number of health technicians per 10,000 people	0.034	0.002
						I_22_ Number of emergency rescue volunteer registered groups per 10,000 people	0.152	0.009
						I_23_ Proportion of water conservancy, environment and public facilities management in employment	0.495	0.028
		C_3_ Enhancement resilience	0.222	T_8_ Risk management	0.438	I_24_ Proportion of the affected population	0.746	0.072
						I_25_ Proportion of direct economic losses caused by disasters to GDP	0.254	0.025
				T_9_ Financial input	0.220	I_26_ Proportion of public security expenditure	0.630	0.031
						I_27_ Proportion of medical and health expenditure	0.370	0.018
				T_10_ Community preparation	0.138	I_28_ Proportion of comprehensive disaster reduction demonstration communities	0.409	0.013
						I_29_ Number of community service agencies per 10,000 population	0.591	0.018
				T_11_ Social recovery	0.204	I_30_ Proportion of the population with basic medical insurance	0.407	0.018
						I_31_ Disposable income per capita	0.593	0.027

**Table 4:** Results of consistency tests.

Hierarchical Objects	CI (average)	CR (average)
Urban resilience for rainstorm and flood disasters	0.008	0.016
Prevention resilience	0.008	0.015
Absorption resilience	0.012	0.014
Enhancement resilience	0.023	0.026
Land layout	0.020	0.022
Monitoring and early warning	0	0
Natural environment	0.006	0.012
Engineering resistance	0.020	0.022
Evacuation and Risk Avoidance	0.015	0.016
Social response	0.066	0.059
Risk management	0	0
Financial input	0	0
Community preparation	0	0
Social recovery	0	0

### 4.4 Resilience calculation

Resilience results are calculated based on the results of data normalization and indicator weights. The results are calculated by the following formula.


$$Re=\frac{\sum \omega_{i}\times X_{i}}{\sum \omega_{i}}$$

In this study, the overall urban resilience to rainstorm and flood disasters is calculated, and the resilience results of sub-items are calculated from two dimensions of resilience abilities and safety factors. Eight categories of resilience results are presented, including one overall resilience result (urban resilience to rainstorm and flood disasters), three categories for the resilience abilities dimension (prevention resilience, absorption resilience and enhancement resilience), and four categories for the safety factors dimension (personnel resilience, facility resilience, environment resilience and management resilience). When calculating the results for different categories of resilience, the indicators associated with that category are considered.

## 5 Results and discussion

### 5.1 Study area

In this study, Beijing was selected as the study area, which is the capital city and the first city proposed the concept of “resilient city” into urban planning in China. In applying the indicator system proposed in this study, the data standardization process requires the comparison of the same indicator data among different study subjects. 16 districts in Beijing become the research subjects. And the data of indicators of the 16 districts in Beijing were collected from the statistics of government sectors as listed in Table 1.

### 5.2 Standard classification

The resilience results of the rainstorm and flood disasters in all districts of Beijing ware calculated based on the weight of all indicators and the standardization result of the indicator data in all districts. According to the assessment model with the dual dimensions of urban disaster resilience, there were respective resilience assessments of the indicators under the correspondent dimension from the perspective of the two dimensions, resilience abilities and safety factors, to conclude the resilience results for the respective dimension of rainstorm and flood disasters in all districts of Beijing.

There were statistics on the results of resilience for rainstorm and flood disasters in all districts of Beijing and the resilience results of respective dimensions. All the eight categories of resilience results of the 16 districts are counted. With the 25%, 50% and 75% quartiles of all the resilience results as reference values, the resilience results were divided into four grades, excellent, good, medium and bad. The value range of each grade was as shown in Table 5.

**Table 5:** Classification of disaster resilience outcome.

Grade	Value Range
Excellent	≥0.600
Good	≥0.500,<0.600
Medium	≥0.400,<0.500
Bad	<0.400

### 5.3 Assessment results of the disaster resilience of all districts

**Fig 3:**
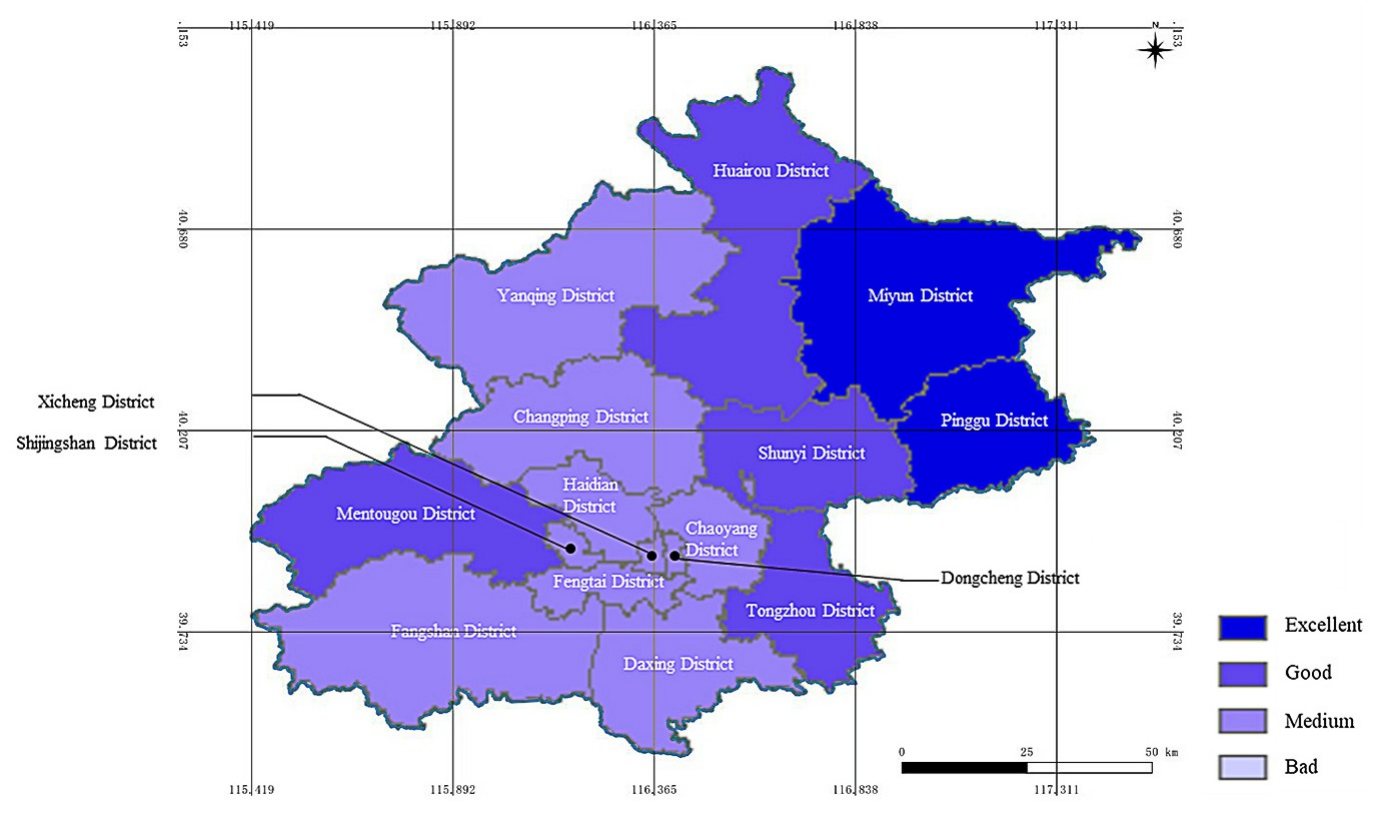
Spatial display of the resilience for rainstorm and flood disasters of all districts of Beijing (The base map was republished from www.tianditu.gov.cn under a CC BY license, with permission from the National Platform for Common Geospatial Information Services).

The assessment results of the resilience to rainstorm and flood disasters in all districts of Beijing are shown in Table 6. There was a spatial display of the results with ArcGIS, as shown in Fig 3.

**Table 6:** The assessment results of the resilience to rainstorm and flood disasters in all districts of Beijing.

District e Name	Dongcheng District	Xicheng District	Chaoyang District	Fengtai District	Shijingshan District	Haidian District	Mentougou District	Fangshan District	Tongzhou District	Shunyi District	Changping District	Daxing District	Huairou District	Pinggu District	Miyun District	Yanqing District
Disaster resilience	0.416	0.405	0.429	0.436	0.475	0.442	0.527	0.478	0.527	0.509	0.473	0.483	0.585	0.641	0.619	0.463

It could be seen that the general resilience to rainstorm and flood disasters in all districts of Beijing was fine. The assessment results of Pinggu District and Miyun District were excellent. The results of Huairou District, Tongzhou District, Mentougou District and Shunyi District were good. The results of the rest of the districts were medium. There was no district with a bad result of resilience to rainstorm and flood disasters. The average value of the assessment result of resilience for rainstorm and flood disasters in 16 districts was 0.494, which was close to the grade of good.

### 5.4 Assessment result of the dimension of resilience abilities in all districts

**Fig 4:**
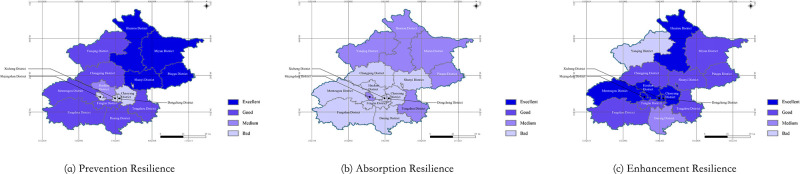
Spatial display of the results of the dimension of resilience abilities of all districts of Beijing (The base map was republished from www.tianditu.gov.cn under a CC BY license, with permission from the National Platform for Common Geospatial Information Services).

There was an analysis of the dimension of resilience abilities, and the results of the prevention resilience, absorption resilience and enhancement resilience in all districts were as shown in Table 7. The spatial display of the results was as shown in Fig 4.

**Table 7:** Assessment results of the dimensions of resilience in Beijing districts.

District Name	Dongcheng District	Xicheng District	Chaoyang District	Fengtai District	Shijingshan District	Haidian District	Mentougou District	Fangshan District	Tongzhou District	Shunyi District	Changping District	Daxing District	Huairou District	Pinggu District	Miyun District	Yanqing District
Prevention resilience	0.225	0.248	0.319	0.525	0.37	0.428	0.561	0.535	0.570	0.604	0.591	0.560	0.649	0.814	0.752	0.593
Absorption resilience	0.386	0.371	0.352	0.281	0.499	0.284	0.366	0.327	0.406	0.341	0.300	0.376	0.410	0.414	0.439	0.413
Enhancement resilience	0.843	0.769	0.766	0.528	0.65	0.705	0.667	0.562	0.595	0.536	0.512	0.462	0.681	0.592	0.585	0.257

The average results of the prevention resilience, absorption resilience and enhancement resilience of all districts of Beijing were 0.522, 0.373 and 0.607. On the whole, the prevention resilience and enhancement resilience in all districts were better, but the absorption resilience was not that ideal. In the aspect of prevention resilience, the results of prevention resilience in 11 districts were in the grade of excellent or good; however, the results in Shijingshan District, Chaoyang District, Xicheng District, Dongcheng District were worse, and they should be further improved. In the aspect of absorption resilience, only the results of the 6 districts were in the grade of a medium, and the results of the rest of the districts were bad. There should be an improvement in the absorption resilience of all districts. For the enhancement resilience, only the result of Daxing District was medium and the one of Yanqing District was bad, while the results of the other 14 districts were excellent or good. It meant that Beijing showed a strongly enhanced resilience on the whole.

### 5.5 Assessment result of the dimension of safety factors of all districts

**Fig 5:**
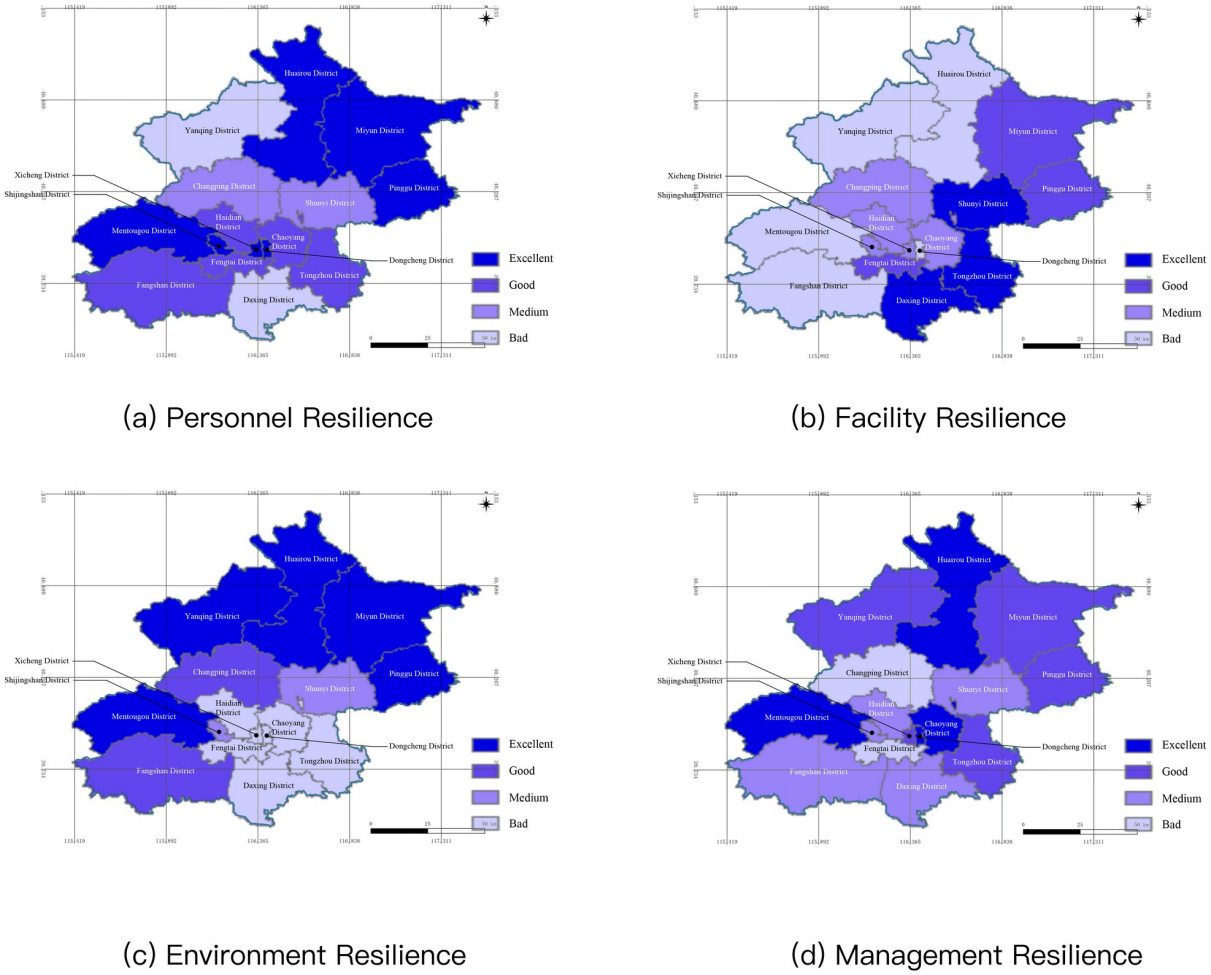
Spatial display of the results of the dimension of safety factors of all districts of Beijing (The base map was republished from www.tianditu.gov.cn under a CC BY license, with permission from the National Platform for Common Geospatial Information Services).

There was an analysis of the dimension of safety factors, and the results of the personnel resilience, facility resilience, ecological resilience and management resilience of all districts were shown in Table 8. The spatial display of the results was as shown in Fig 5.

**Table 8:** Assessment results of security factors dimensions of Beijing districts.

District Name	Dongcheng District	Xicheng District	Chaoyang District	Fengtai District	Shijingshan District	Haidian District	Mentougou District	Fangshan District	Tongzhou District	Shunyi District	Changping District	Daxing District	Huairou District	Pinggu District	Miyun District	Yanqing District
Personnel resilience	0.672	0.364	0.196	0.697	0.672	0.364	0.196	0.697	0.672	0.364	0.196	0.697	0.672	0.364	0.196	0.697
Facility resilience	0.69	0.418	0.113	0.544	0.69	0.418	0.113	0.544	0.69	0.418	0.113	0.544	0.69	0.418	0.113	0.544
Environment resilience	0.588	0.469	0.154	0.662	0.588	0.469	0.154	0.662	0.588	0.469	0.154	0.662	0.588	0.469	0.154	0.662
Management resilience	0.534	0.536	0.303	0.261	0.534	0.536	0.303	0.261	0.534	0.536	0.303	0.261	0.534	0.536	0.303	0.261

The average results of the personnel resilience, facility resilience, cological resilience and management resilience of all districts were 0.556, 0.473, 0.475 and 0.509. The assessment results of the dimension of safety factors showed different features in the spatial distribution. For personnel resilience, there were excellent results in the districts in the northeast and central areas of Beijing. The reason for the strong personnel resilience in the northeast area was the lower population density and small density of disaster bearing bodies, and the reason why there was strong personnel resilience in the central area was the strong self-organization ability of residents and relatively complete supporting services. Given the facility’s resilience, there was a better result in the districts in the east and central areas of Beijing while there was a worse result in the districts in the western areas, which should be owed to the distribution of overall water conservancy, meteorology and disaster prevention facilities in Beijing. As for environmental resilience, there was a better overall situation in the districts in the north and west areas of Beijing; while there was a worse overall situation in the districts in the central and southeast areas of Beijing. It was caused by the natural environment in the city. The north and west areas had a better natural foundation with a higher proportion of blue and green space. In the management resilience, the results of all districts were medium or higher except for Fengtai District and Changping District whose results were mainly medium. There were relatively smaller differences among the results of all districts, which should be thanked for the common experience in the safety management systems and mechanisms in the governments of all districts. The difference was mainly related to the risk background and financial input of each district.

### 5.6 Comparison between the results of central districts and the ones of non-central districts

Dongcheng District, Xicheng District, Chaoyang District, Haidian District, Fengtai District and Shijingshan District, these 6 districts were the central ones in Beijing. And the rest of them were the non-central districts. Thus, there was a calculation of all assessment results of the resilience for rainstorm and flood disasters in the central districts and the non-central districts to gain the average results, and the results were as shown in Table 9.

**Table 9:** Comparison of average resilience results between central and non-central urban districts in Beijing.

Result classification	Result kind	The average result of central urban districts	Average results of non-central urban districts
Disaster resilience	Disaster resilience	0.434	0.530
Resilience ability	Prevention resilience	0.353	0.623
	Absorption resilience	0.362	0.379
	Enhancement resilience	0.710	0.545
Safety factors	Personnel resilience	0.616	0.520
	Facility resilience	0.442	0.491
	Environment resilience	0.257	0.607
	Management resilience	0.516	0.505

It could be seen from the table that the overall situation of the resilience for rainstorm and flood disasters in central districts was worse than the one of the non-central districts, and the reasons could be compared and analyzed through the assessment results of all resilience abilities and safety factors. In the resilience ability, the prevention resilience in the central districts was far lower than that of the non-central districts since the central districts had a higher difficulty in the prevention of rainstorm and flood disasters due to the high density of disaster-bearing bodies, more complex urban systems and higher protection requirements. At the same time, the enhancement resilience in the central districts was better than that of the non-central districts since there was a more developed economy and a better risk transfer and post-disaster recovery system in the central districts. In the aspect of safety factors, the personnel resilience in the central districts was higher than in the non-central districts since there was a better disaster rescue and relief system in the central districts. However, the environmental resilience in the central districts was far lower than that of the non-central districts, which was related to the foundation of the natural environment and showed the negative influence of the high urbanization on disaster resilience.

### 5.7 Comparison with similar studies

The relationship between risk and resilience is vague. Different researchers have discussed the relationship between resilience and risk from a doctrinal perspective and have presented different opinions. Resilience could be seen as the opposite of risk, or a management approach to risk, or an interconnected but different concept with risk, or a completely separate concept to risk [28,49–52]. Instead of exploring the relationship between risk and resilience conceptually, this study verified the relationship between the two concepts from the perspective of empirical research. In 2022, Beijing published an urban waterlogging risk map to identify urban high-risk points under rainstorm and flood disasters [53]. The density of risk points in each district is compared with the resilience results of storm flooding hazards obtained from this study and ranked from highest to lowest resilience, as shown in Table 10.

**Table 10:** Comparison of resilience and density of inundation points.

District	Resilience for rainstorm and flood disasters	Density of inundation points (pcs/km2)
Pinggu District	0.641	0
Miyun District	0.619	0
Huairou District	0.585	0
Tongzhou District	0.527	0.016
Mentougou District	0.527	0
Shunyi District	0.509	0
Daxing District	0.483	0
Fangshan District	0.478	0
Shijingshan District	0.475	0.071
Changping District	0.473	0.024
Yanqing District	0.463	0
Haidian District	0.442	0.043
Fengtai District	0.436	0.102
Chaoyang District	0.429	0.028
Dongcheng District	0.416	0.095
Xicheng District	0.405	0.08

As can be seen from the table, districts without high risk points also generally received higher resilience measurement results in this study, which also indicates that risk is an important driver of resilience. At the same time, the resilience results of each zone are not exactly opposite to the density of high-risk points, which also reflects the difference between resilience studies and risk studies, that is, resilience studies also consider the influence of the system’s own abilities and component factors.

### 5.8 Discussion

The following assessment conclusions can be drawn from above assessment process. According to the assessment results, the overall situation of the resilience to rainstorm and flood disasters in all districts of Beijing was fine, which showed the achievements gained by Beijing in urban disaster risk prevention and control and resilient city construction. Among the prevention, absorption and enhancement of the reliance abilities, the overall situation of the prevention resilience and enhancement resilience in all districts of Beijing was better, while there was a huge space for improvement in the absorption resilience. A comprehensive improvement of disaster emergency response capability by improving the regulation and storage of natural space, upgrading the engineering fortification standard, strengthening the ability of emergency rescue and refuge, and increasing social disaster relief preparation would be the key link for all districts of Beijing to enhance the resilience for rainstorm and flood disasters. Due to the natural location, and social and economic development, all districts had different situations in the resilience of personnel, facility, environment and management. In General, the highly developed central districts impose a negative impact on the prevention and control of rainstorm and flood disasters. The average resilience for rainstorm and flood disasters in central districts was worse than that in the non-central districts, so there should be further attention on the improvement of disaster adaptability of the natural environment and the built environment.

In addition to the general resilience assessment result (urban resilience for rainstorm and flood disasters) of each research subject like the traditional practice, this paper also give multi-dimensional resilience assessment results according to the categorization of resilience abilities and safety factors. This approach allows for a more intuitive and clearer understanding of the causes of the general resilience assessment result of each subject. It will be more helpful for clarifying the strengths and weaknesses of each research subject, and thus giving directions and suggestions for improving resilience. It is worth noting that the assessment model with the dual dimensions of urban disaster resilience proposed in this paper is a generalized model, which can be used not only for rainstorm and flood disasters, but also other types of disasters. The assessment index system of urban resilience for rainstorm and flood disasters proposed in this study could be seen as a specific application based on this model. When this model is used in the research on other types of disasters, the two-dimensional framework can be used to further construct the assessment system combining the characteristics of the disaster under study and the research subjects.

## 6 Conclusion

The paper suggests an assessment model with the dual dimensions of urban disaster resilience with the dimensions of resilience abilities and safety factors. And it starts from the three resilience abilities, including prevention resilience, absorption resilience and enhancement resilience to screen 31 specific assessment indicators according to the four safety factors, including personnel, facility, environment and management, with the combination of the demand for urban disaster prevention and emergency management in China, to build up an assessment index system of urban resilience for rainstorm and flood disasters. Ten experts are invited to confirm the indicator weight with the analytic hierarchy process. Furthermore, with Beijing as a study object, the paper assesses the resilience for rainstorm and flood disasters of 16 districts of Beijing and the respective resilience results for the resilience abilities and the safety factors. The result shows that the overall situation of resilience for rainstorm and flood disasters in all districts of Beijing is fine, while all districts have their situation in various resilience abilities and safety factors. Thus, in the next stage, there should be further improvement of the urban resilience for rainstorm and flood disasters with the key point of improving the absorption resilience. And there should be an improvement in the resilience to rainstorm and flood disasters in the central districts from disaster prevention and environmental improvement. It is worth mentioning that the assessment model with the dual dimensions proposed in this paper is of general significance and can be applied to resilience assessments of other disasters, not limited to rainstorm and flood disasters. The proposed asssessment index system of urban resilience for rainstorm and flood disasters also has strong generalizability for China’s cities due to the channel of government management statistics. It should be noted that the results obtained in this study are relative results for the comparison within the 16 districts of Beijing, and the method can also be applied to different cities to obtain comparative results of resilience between different cities. In addition, due to the limitation of the statistical channel and update time of the data, the data year taken in this study is a single year data, and the trajectory of city resilience improvement can be obtained by comparing the results between different years in further study.

## Supporting information

S1 File (DOCX)
