# Supplementary material for: A research on urban disaster resilience assessment system for rainstorm and flood disasters: A case study of Beijing
Source: PLoS One. 2023 Oct 26;18(10):e0291674. doi: 10.1371/journal.pone.0291674 (PMC10602262; doi:10.1371/journal.pone.0291674)
Supplement: S1 File — (DOCX) [file pone.0291674.s001.docx]

**Specific Information on Where and How to Access the Data Sources**

The specific information on where and how to access the statistical yearbook and government documentation mentioned in data availability statement are listed as below.

Table 1 Original Data Source URL of Indicators

| **Indicator** | **Data Source Publication or URL** |
| --- | --- |
| I_1_ Land Development Intensity | Publication: Beijing Regional Statistical Yearbook 2021 (ISBN: 9787503795817)  URL: https://nj.tjj.beijing.gov.cn/nj/qxnj/2021/zk/e/indexce.htm |
| I_2_ Proportion of Water and Soil Loss Control Area | URL: http://swj.beijing.gov.cn/zwgk/swtjnj/202304/t20230413_3032357.html |
| I_3_ Proportion of Water Area and Water Conservancy Facilities | Publication: Beijing Regional Statistical Yearbook 2021 (ISBN: 9787503795817)  URL: https://nj.tjj.beijing.gov.cn/nj/qxnj/2021/zk/e/indexce.htm |
| I_4_ Proportion of land for transportation facilities | Publication: Beijing Regional Statistical Yearbook 2021 (ISBN: 9787503795817)  URL: https://nj.tjj.beijing.gov.cn/nj/qxnj/2021/zk/e/indexce.htm |
| I_5_ Early warning amount of sudden disastrous weather | URL: http://bj.cma.gov.cn/zfxxgk/zwgk/czzj/bmyjsxx/ |
| I_6_ Number of Disaster Information Officers per 10,000 People | URL: http://yjglj.beijing.gov.cn/art/2021/12/29/art_9100_622448.html |
| I_7_ Mobile phone penetration | URL：  (1)http://www.bjdch.gov.cn/n2001806/n2917385/n2917389/c11397942/part/11397947.pdf  (2)https://www.bjxch.gov.cn/xcsj/tjnj/2021tjnj.html  (3)https://inds.cnki.net/knavi/yearbook/Detail/YJGY/YBJCY?NO=N2019080014&uid=WEEvREcwSlJHSldSdmVqMDh6c3VFeXBRU1JjRm1WcERZOEY5QnkrTEtIcz0=$9A4hF_YAuvQ5obgVAqNKPCYcEjKensW4ggI8Fm4gTkoUKaID8j8gFw!!  (4)https://data.cnki.net/yearBook/single?id=N2022010138  (5)http://www.bjsjs.gov.cn/gongkai/zwgkpd/zdly_1960/ndxx/202204/P020220425590905195990.pdf  (6)https://data.cnki.net/yearBook/single?id=N2022030029  (7)https://data.cnki.net/yearBook/single?id=N2021120166  (8)https://data.cnki.net/yearBook/single?id=N2022070084  (9)https://data.cnki.net/yearBook/single?id=N2022010137  (10)http://www.bjshy.gov.cn/web/zwgk/tjxx/ndsj/1061327/index.html  (11)https://data.cnki.net/yearBook/single?id=N2022070104  (12)https://data.cnki.net/yearBook/single?id=N2021120025  (13)http://www.bjhr.gov.cn/zwgk/sj/tjnj/202112/P020211214480721930520.xls  (14)http://www.bjpg.gov.cn/pgqrmzf/zwxx0/sjxz/index.html  (15)http://www.bjmy.gov.cn/module/download/downfile.jsp?classid=1&filename=3f1057b03aae4979ae7a0160dcc2d752.pdf  (16)http://www.bjyq.gov.cn/yanqing/zbm/1718459/1880121/index.shtml |
| I_8_ Annual rainfall | URL: https://www.tianqi24.com/historycity |
| I_9_ Reservoir density | URL: http://swj.beijing.gov.cn/zwgk/swtjnj/202304/t20230413_3032357.html |
| I_10_ Greening coverage | Publication: Beijing Regional Statistical Yearbook 2021 (ISBN: 9787503795817)  URL: https://nj.tjj.beijing.gov.cn/nj/qxnj/2021/zk/e/indexce.htm |
| I_11_ Pump station density | URL: http://swj.beijing.gov.cn/zwgk/swtjnj/202304/t20230413_3032357.html |
| I_12_ Comprehensive rainwater utilization capacity | URL: http://swj.beijing.gov.cn/zwgk/swtjnj/202304/t20230413_3032357.html |
| I_13_ Proportion of urban built-up areas that can absorb and utilize 70% of rainfall locally | URL: http://swj.beijing.gov.cn/zwgk/swtjnj/202304/t20230413_3032357.html |
| I_14_ Dike density | URL: http://swj.beijing.gov.cn/zwgk/swtjnj/202304/t20230413_3032357.html |
| I_15_ Road area per capita | URL：  (1)http://www.bjdch.gov.cn/n2001806/n2917385/n2917389/c11397942/part/11397947.pdf  (2)https://www.bjxch.gov.cn/xcsj/tjnj/2021tjnj.html  (3)https://inds.cnki.net/knavi/yearbook/Detail/YJGY/YBJCY?NO=N2019080014&uid=WEEvREcwSlJHSldSdmVqMDh6c3VFeXBRU1JjRm1WcERZOEY5QnkrTEtIcz0=$9A4hF_YAuvQ5obgVAqNKPCYcEjKensW4ggI8Fm4gTkoUKaID8j8gFw!!  (4)https://data.cnki.net/yearBook/single?id=N2022010138  (5)http://www.bjsjs.gov.cn/gongkai/zwgkpd/zdly_1960/ndxx/202204/P020220425590905195990.pdf  (6)https://data.cnki.net/yearBook/single?id=N2022030029  (7)https://data.cnki.net/yearBook/single?id=N2021120166  (8)https://data.cnki.net/yearBook/single?id=N2022070084  (9)https://data.cnki.net/yearBook/single?id=N2022010137  (10)http://www.bjshy.gov.cn/web/zwgk/tjxx/ndsj/1061327/index.html  (11)https://data.cnki.net/yearBook/single?id=N2022070104  (12)https://data.cnki.net/yearBook/single?id=N2021120025  (13)http://www.bjhr.gov.cn/zwgk/sj/tjnj/202112/P020211214480721930520.xls  (14)http://www.bjpg.gov.cn/pgqrmzf/zwxx0/sjxz/index.html  (15)http://www.bjmy.gov.cn/module/download/downfile.jsp?classid=1&filename=3f1057b03aae4979ae7a0160dcc2d752.pdf  (16)http://www.bjyq.gov.cn/yanqing/zbm/1718459/1880121/index.shtml |
| I_16_ Road traffic index | Publication: Beijing Traffic Development Report 2021 (ISBN: 9787520195560)  URL: https://www.pishu.com.cn/skwx_ps/bookdetail?SiteID=14&ID=13638599 |
| I_17_ Number of private cars per 100 households | Publication: Beijing Regional Statistical Yearbook 2021 (ISBN: 9787503795817)  URL: https://nj.tjj.beijing.gov.cn/nj/qxnj/2021/zk/e/indexce.htm |
| I_18_ Area of refuge per capita | URL: http://yjglj.beijing.gov.cn/col/col8708/index.html |
| I_19_ Permanent population density | Publication: Beijing Regional Statistical Yearbook 2021 (ISBN: 9787503795817)  URL: https://nj.tjj.beijing.gov.cn/nj/qxnj/2021/zk/e/indexce.htm |
| I_20_ Proportion of young and old people | Publication: Beijing Regional Statistical Yearbook 2021 (ISBN: 9787503795817)  URL: https://nj.tjj.beijing.gov.cn/nj/qxnj/2021/zk/e/indexce.htm |
| I_21_ Number of health technicians per 10,000 people | Publication: Beijing Regional Statistical Yearbook 2021 (ISBN: 9787503795817)  URL: https://nj.tjj.beijing.gov.cn/nj/qxnj/2021/zk/e/indexce.htm |
| I_22_ Number of emergency rescue volunteer registered groups per 10,000 people | URL: https://www.bv2008.cn/app/org/list.php |
| I_23_ Proportion of water conservancy, environment and public facilities management in employment | URL：  (1)http://www.bjdch.gov.cn/n2001806/n2917385/n2917389/c11397942/part/11397947.pdf  (2)https://www.bjxch.gov.cn/xcsj/tjnj/2021tjnj.html  (3)https://inds.cnki.net/knavi/yearbook/Detail/YJGY/YBJCY?NO=N2019080014&uid=WEEvREcwSlJHSldSdmVqMDh6c3VFeXBRU1JjRm1WcERZOEY5QnkrTEtIcz0=$9A4hF_YAuvQ5obgVAqNKPCYcEjKensW4ggI8Fm4gTkoUKaID8j8gFw!!  (4)https://data.cnki.net/yearBook/single?id=N2022010138  (5)http://www.bjsjs.gov.cn/gongkai/zwgkpd/zdly_1960/ndxx/202204/P020220425590905195990.pdf  (6)https://data.cnki.net/yearBook/single?id=N2022030029  (7)https://data.cnki.net/yearBook/single?id=N2021120166  (8)https://data.cnki.net/yearBook/single?id=N2022070084  (9)https://data.cnki.net/yearBook/single?id=N2022010137  (10)http://www.bjshy.gov.cn/web/zwgk/tjxx/ndsj/1061327/index.html  (11)https://data.cnki.net/yearBook/single?id=N2022070104  (12)https://data.cnki.net/yearBook/single?id=N2021120025  (13)http://www.bjhr.gov.cn/zwgk/sj/tjnj/202112/P020211214480721930520.xls  (14)http://www.bjpg.gov.cn/pgqrmzf/zwxx0/sjxz/index.html  (15)http://www.bjmy.gov.cn/module/download/downfile.jsp?classid=1&filename=3f1057b03aae4979ae7a0160dcc2d752.pdf  (16)http://www.bjyq.gov.cn/yanqing/zbm/1718459/1880121/index.shtml |
| I_24_ Proportion of the affected population | URL: http://yjglj.beijing.gov.cn/art/2021/12/29/art_9100_622448.html |
| I_25_ Proportion of direct economic losses caused by disasters to GDP | URL: http://yjglj.beijing.gov.cn/art/2021/12/29/art_9100_622448.html |
| I_26_ Proportion of public security expenditure | URL：  (1)http://www.bjdch.gov.cn/n2001806/n2917385/n2917389/c11397942/part/11397947.pdf  (2)https://www.bjxch.gov.cn/xcsj/tjnj/2021tjnj.html  (3)https://inds.cnki.net/knavi/yearbook/Detail/YJGY/YBJCY?NO=N2019080014&uid=WEEvREcwSlJHSldSdmVqMDh6c3VFeXBRU1JjRm1WcERZOEY5QnkrTEtIcz0=$9A4hF_YAuvQ5obgVAqNKPCYcEjKensW4ggI8Fm4gTkoUKaID8j8gFw!!  (4)https://data.cnki.net/yearBook/single?id=N2022010138  (5)http://www.bjsjs.gov.cn/gongkai/zwgkpd/zdly_1960/ndxx/202204/P020220425590905195990.pdf  (6)https://data.cnki.net/yearBook/single?id=N2022030029  (7)https://data.cnki.net/yearBook/single?id=N2021120166  (8)https://data.cnki.net/yearBook/single?id=N2022070084  (9)https://data.cnki.net/yearBook/single?id=N2022010137  (10)http://www.bjshy.gov.cn/web/zwgk/tjxx/ndsj/1061327/index.html  (11)https://data.cnki.net/yearBook/single?id=N2022070104  (12)https://data.cnki.net/yearBook/single?id=N2021120025  (13)http://www.bjhr.gov.cn/zwgk/sj/tjnj/202112/P020211214480721930520.xls  (14)http://www.bjpg.gov.cn/pgqrmzf/zwxx0/sjxz/index.html  (15)http://www.bjmy.gov.cn/module/download/downfile.jsp?classid=1&filename=3f1057b03aae4979ae7a0160dcc2d752.pdf  (16)http://www.bjyq.gov.cn/yanqing/zbm/1718459/1880121/index.shtml |
| I_27_ Proportion of medical and health expenditure | URL：  (1)http://www.bjdch.gov.cn/n2001806/n2917385/n2917389/c11397942/part/11397947.pdf  (2)https://www.bjxch.gov.cn/xcsj/tjnj/2021tjnj.html  (3)https://inds.cnki.net/knavi/yearbook/Detail/YJGY/YBJCY?NO=N2019080014&uid=WEEvREcwSlJHSldSdmVqMDh6c3VFeXBRU1JjRm1WcERZOEY5QnkrTEtIcz0=$9A4hF_YAuvQ5obgVAqNKPCYcEjKensW4ggI8Fm4gTkoUKaID8j8gFw!!  (4)https://data.cnki.net/yearBook/single?id=N2022010138  (5)http://www.bjsjs.gov.cn/gongkai/zwgkpd/zdly_1960/ndxx/202204/P020220425590905195990.pdf  (6)https://data.cnki.net/yearBook/single?id=N2022030029  (7)https://data.cnki.net/yearBook/single?id=N2021120166  (8)https://data.cnki.net/yearBook/single?id=N2022070084  (9)https://data.cnki.net/yearBook/single?id=N2022010137  (10)http://www.bjshy.gov.cn/web/zwgk/tjxx/ndsj/1061327/index.html  (11)https://data.cnki.net/yearBook/single?id=N2022070104  (12)https://data.cnki.net/yearBook/single?id=N2021120025  (13)http://www.bjhr.gov.cn/zwgk/sj/tjnj/202112/P020211214480721930520.xls  (14)http://www.bjpg.gov.cn/pgqrmzf/zwxx0/sjxz/index.html  (15)http://www.bjmy.gov.cn/module/download/downfile.jsp?classid=1&filename=3f1057b03aae4979ae7a0160dcc2d752.pdf  (16)http://www.bjyq.gov.cn/yanqing/zbm/1718459/1880121/index.shtml |
| I_28_ Proportion of comprehensive disaster reduction demonstration communities | URL:  (1)<http://yjglj.beijing.gov.cn/art/2020/6/29/art_8994_617892.html>  (2)http://yjglj.beijing.gov.cn/art/2021/1/27/art_8994_618016.html |
| I_29_ Number of community service agencies per 10,000 population | Publication: Beijing Regional Statistical Yearbook 2021 (ISBN: 9787503795817)  URL: https://nj.tjj.beijing.gov.cn/nj/qxnj/2021/zk/e/indexce.htm |
| I_30_ Proportion of the population with basic medical insurance | Publication: Beijing Regional Statistical Yearbook 2021 (ISBN: 9787503795817)  URL: https://nj.tjj.beijing.gov.cn/nj/qxnj/2021/zk/e/indexce.htm |
| I_31_ Disposable income per capita | Publication: Beijing Regional Statistical Yearbook 2021 (ISBN: 9787503795817)  URL: https://nj.tjj.beijing.gov.cn/nj/qxnj/2021/zk/e/indexce.htm |
